# A Clustering Scheme Based on the Binary Whale Optimization Algorithm in FANET

**DOI:** 10.3390/e24101366

**Published:** 2022-09-27

**Authors:** Yonghang Yan, Xuewen Xia, Lingli Zhang, Zhijia Li, Chunbin Qin

**Affiliations:** 1 School of Computer and Information Engineering, Henan University, Kaifeng 475004, China; 2 Henan Province Engineering Research Center of Spatial Information Processing, Henan University, Kaifeng 475004, China; 3 Beijing Aerospace Automatic Control Institute, Beijing 100854, China; 4 School of Artificial Intelligence, Henan University, Kaifeng 475004, China

**Keywords:** FANET, UAV clusters, clustering, binary whale optimization algorithm (BWOA)

## Abstract

With the continuous development of Unmanned Aerial Vehicle (UAV) technology, UAVs are widely used in military and civilian fields. Multi-UAV networks are often referred to as flying ad hoc networks (FANET). Dividing multiple UAVs into clusters for management can reduce energy consumption, maximize network lifetime, and enhance network scalability to a certain extent, so UAV clustering is an important direction for UAV network applications. However, UAVs have the characteristics of limited energy resources and high mobility, which bring challenges to UAV cluster communication networking. Therefore, this paper proposes a clustering scheme for UAV clusters based on the binary whale optimization (BWOA) algorithm. First, the optimal number of clusters in the network is calculated based on the network bandwidth and node coverage constraints. Then, the cluster heads are selected based on the optimal number of clusters using the BWOA algorithm, and the clusters are divided based on the distance. Finally, the cluster maintenance strategy is set to achieve efficient maintenance of clusters. The experimental simulation results show that the scheme has better performance in terms of energy consumption and network lifetime compared with the BPSO and K-means-based schemes.

## 1. Introduction

In recent years, UAVs have attracted widespread attention as a rapidly developing emerging technology in academic research, military applications, and civil use. UAVs have the advantages of small size, low-cost operation, rapid deployment, easy access to hazardous areas, flexibility, scalability, etc. However, due to the limited energy and computing power of a single UAV, it is impossible to ensure optimal working conditions at all times, while close collaboration between multiple UAVs to form a UAV cluster can be used to accomplish various tasks in complex and special environments. Therefore, it has gradually become an important form of current UAV combat applications.

In a FANET, all UAVs establish an ad hoc network, and only a subset of the UAVs connect to the infrastructure; the others use intermediate UAVs to communicate with the ground controller in a multi-hop way [1]. FANET is widely used in many fields, such as emergency communications [2], battlefield applications, target tracking [3], timely recovery services after natural disasters, wildfire detection and surveillance, relay for ad hoc networks, cargo transportation and delivery [4], search and rescue operations [5], and other areas.

**Figure 1:**
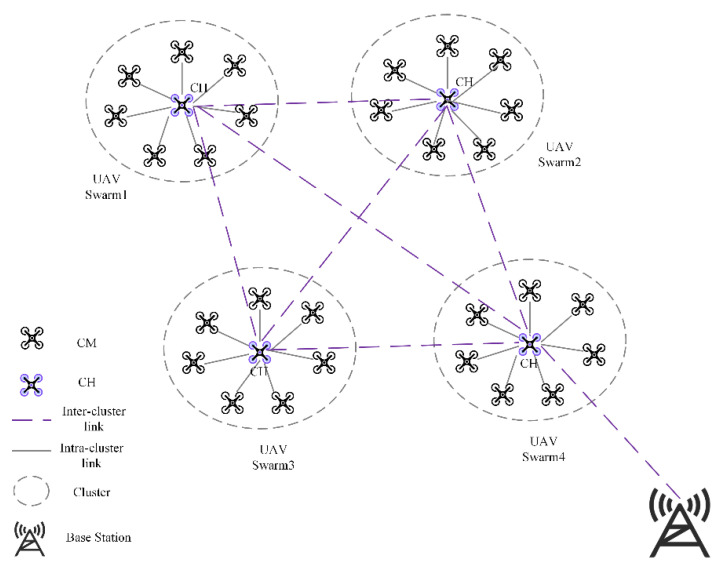
FANET Communication Architecture based on Clustering.

The high mobility of the UAV nodes in FANET makes it more frequent to enter and exit the network, which causes difficulties in the establishment and maintenance of the network and makes it difficult to control and manage the UAVs efficiently as their scale increases. As shown in Figure 1, dividing the network into clusters can help solve the above problems. The division process is based on different parameters, and UAVs are divided into different cluster groups that can communicate directly with each other and share channels and resources within the effective communication range of the nodes. Based on different election parameters, the UAV with the highest performance is selected as the cluster head (CH), and the rest of the UAVs in the group are cluster members (CM). The CH nodes are responsible for inter-cluster intra-cluster data forwarding in the UAV network, and the nodes transmit packets to the CH, which forwards them to the destination node or base station. In this way, the number of control packets will be reduced. However, the communication burden of CH will be increased because it needs to transmit data between clusters and manage CMs. Therefore, the division of clusters and the selection of CHs, as well as efficient cluster management strategies, are essential to achieve reliable communication and improve network performance in a hierarchical network.

To reasonably utilize the network resources, this paper first calculates the optimal number of clusters based on the network bandwidth and the coverage capacity of UAV nodes. To better manage UAV clusters, this paper proposes a UAV cluster clustering scheme based on the binary whale optimization (BWOA) algorithm to avoid frequent cluster reconfiguration and make the cluster lifetime longer by selecting more stable cluster heads and setting efficient cluster maintenance strategies, thus reducing the network overhead. For the routing problem in clusters, the design of inter-cluster and intra-cluster UAV nodes communication routes are considered separately to make the inter-node load balancing and routing overhead smaller, thus maximizing the network throughput.

The rest of the paper is organized as follows: Section 2 presents the existing work on the cluster clustering scheme in UAV communication. Section 3 presents some preparatory work. Section 4 describes the clustering scheme proposed in this paper in detail, including the calculation of the optimal number of clusters, cluster partitioning, and management mechanism. Simulation results and performance analysis are given in Section 5. Section 6 provides the conclusion and the outlook for future work.

## 2. Related Work

Unlike mobile ad hoc networks (MANET) and vehicular ad hoc networks (VANET), FANETs are characterized by high mobility, limited energy resources, frequent topology changes, and unstable wireless links; therefore, traditional clustering schemes may not apply to the highly dynamic application scenarios of UAVs. In recent years, several algorithms for FANETs have been proposed to solve the clustering problem.

Raza A [6] et al. proposed a link-based energy-aware clustering (EALC) model. The K-means density clustering algorithm is used in EALC to select the optimal cluster head considering the node energy level and distance to its neighbors and to determine the cluster number size considering the communication range of the cluster head to improve the cluster lifetime and reduce the overhead. The literature [7] proposed a mobile and location-aware stable clustering (MLSC) mechanism to find the optimal cluster size based on the maximum coverage probability of cluster heads to minimize the network overhead and then use a distance-based K-means clustering algorithm to select stable cluster heads at the optimal location to improve the performance and reliability of UAV networks with limited resources, but the scheme design does not take into account the UAV energy level, and clusters may require frequent maintenance.

A simple area division cluster-based algorithm (SAD-CA), which is a cluster structure at deployment, has been proposed in the literature [8] as a stable and reliable network architecture with low energy consumption and overhead for application scenarios such as surveillance and reconnaissance. To address the problems posed by the high-speed mobility of UAVs and dynamically changing topologies to communication stability in FANETs, an improved weighted and location-based clustering (IWLC) scheme has been proposed in [9], which first uses a location-based K-means++ clustering algorithm to construct initial UAV clusters. Subsequently, a weighted summation-based cluster head selection algorithm has been proposed, which uses residual energy ratio, adaptive node degree, relative mobility, and average distance as selection criteria. This scheme improves the performance of FANETs under network resource constraints.

Some researchers have used swarm intelligence (SI) algorithms to design clustering schemes. A bio-inspired clustering scheme (BICSF) based on a hybrid mechanism of glowworm swarm optimization (GSO) and krill herd (KH) was proposed in the literature [10] to address the problem of routing instability due to limited energy resources and mobility of UAVs. BICSF uses a glowworm optimization algorithm (GSO) to elect cluster heads using the residual energy and fluorescein levels of UAVs and a krill herd (KH) intelligent algorithm to achieve efficient cluster management. Khan, A. [11] et al. proposed an intelligent cluster routing scheme (CRSF) for fly ad hoc networks to solve the communication problem due to UAV maneuverability. The optimal cluster head is selected based on UAV location and residual energy, and a cluster management mechanism inspired by Moth Flame Optimization (MFO) is proposed.

Arafat M Y and Moh S [12] proposed an energy-efficient swarm intelligent clustering (SIC) algorithm based on the PSO algorithm, which considers location information, inter-cluster distance, intra-cluster distance, and residual energy to cluster and select cluster heads of nodes, minimizes network energy consumption, and extends the network lifetime. Bio-inspired clustering (BIC) based algorithm is proposed in the literature [13], BIC uses the hybrid gray wolf optimization (HGWO) algorithm for optimization of cluster formation and cluster head selection to reduce energy consumption by considering the number of clusters, cluster size, cluster stability and the number of transmissions. A new dynamic clustering mechanism for load balancing is proposed in the literature [14], based on Political Optimizer (PO) algorithm for clustering and Shannon entropy function to solve the cluster fault tolerance and traffic overload problems for efficient packet transmission and load balancing.

Because of the characteristics of self-organization, good scalability, and high robustness of the population intelligence optimization algorithm, this paper uses the population intelligence optimization algorithm to select CH. The main contributions are as follows:

- To reasonably make full use of network resources, this paper calculates the optimal number of clusters to be divided in the network based on the network bandwidth and node coverage capacity to maximize the utilization of network resources.
- A clustering scheme based on the binary whale optimization algorithm (CSBWOA) is proposed. Since the whale optimization algorithm has the advantages of simple operation, few parameters to adjust, and a strong ability to jump out of the local optimum, the discrete binary whale optimization (BWOA) algorithm is used for CH selection in this paper. This algorithm selects cluster heads based on the fitness functions of residual energy, intra-cluster distance, and inter-cluster distance to make the cluster lifetime longer. Multi-hop routing is used for communication between clusters to reduce energy consumption.

Table 1 summarizes and compares the scheme of this paper with other clustering schemes. The scheme in this paper integrates distance, energy, and load balancing of clustering in CH selection. It compares performance in terms of cluster building time, cluster lifetime, energy consumption, and node survival rate.

## 3. System Model

### 3.1. Network Model

Suppose *N* UAVs are randomly deployed in a network area of 2000 m × 2000 m × 500 m, UAVs have unique identification IDs, node locations are (x, y, z), base station location coordinates are (0, 0, 0), all UAV nodes have the same computing power, communication power, and initial energy $E_{i}$, each UAV has a sensing radius *R* of 250 m and a communication radius *r* of 300 m. Now we need to divide *N* UAVs into *K* UAV clusters for lower energy consumption and higher performance, and $U=\{{UAV}_{1},{UAV}_{2},\ldots ,{UAV}_{i},\ldots ,{UAV}_{N}\}$ is the set of all UAV nodes, $C=\{{CH}_{1},{CH}_{2},\ldots ,{CH}_{j},\ldots ,{CH}_{k}\}$ is the set of all CH nodes.

### 3.2. Energy Model

The energy consumption of the UAV in FANET is mainly reflected in the energy required to fly and hover the UAV, the energy consumed to communicate with other UAVs, and the energy required by the sensors on the UAV.

(1) $$E_{T}=E_{H}+E_{F}+E_{C}+E_{s}$$

The flight and hover energy consumption of the UAV is referred to [15], and it is calculated as shown in Equation (4). The UAV hover power is calculated as follows:

(2) $$PH=\sqrt{\frac{{\left(m_{u}g\right)}^{3}}{2\pi r_{w}^{2}n_{w}\rho_{a}}}$$

where *m_u_* is the UAV mass, *g* is the acceleration of gravity, $r_{w}$ and $n_{w}$ denote the radius and number of wings, respectively, and $\rho_{a}$ is the air density.

The UAV flight power is calculated as follows:

(3) $$P_{F}=(P_{\max}-P_{H})\frac{v_{u}(t)}{v_{\max}}$$

where $v_{\max}$ is the maximum UAV flight speed, *v_u_* (*t*) is the UAV flight speed during time gap *t*, and *P_max_* is the UAV flight power when the speed is $v_{\max}$.

The UAV hovering and flight energy consumption:

(4) $$\{\begin{array}{c} E_{H}=P_{H}T_{H}\\ E_{F}=\int_{0}^{T_{F}}P_{F}dt \end{array}$$

where *T_H_* and *T_F_* represent the hovering and flying time, respectively.

The energy consumption for data transmission between UAVs is referenced in [16], and the channel model is selected based on the distance between nodes. The energy consumed by a node to transmit *l*-bit data at a distance *d* is calculated as follows:

(5) $$E_{TX}(l,d)=\{\begin{array}{c} \begin{array}{cc} {lE}_{elec}+l\varepsilon_{fs}d^{2} & d<d_{t} \end{array}\\ \begin{array}{cc} {lE}_{elec}+l\varepsilon_{amp}d^{4} & d\geq d_{t} \end{array} \end{array}$$

where $E_{elec}$ denotes the energy consumption for transmitting *l* bits of data, $\varepsilon_{fs}$ and indicates the energy consumption parameters of the power amplifier in the free-space model and the multipath fading model, respectively, and *d_t_* is the distance threshold, calculated as follows:

(6) $$d_{t}=\sqrt{\frac{\varepsilon_{fs}}{\varepsilon_{amp}}}$$

The energy consumed to receive *l* bit data is calculated as follows:

(7) $$E_{RX}(l)=l\times E_{elec}$$

### 3.3. WOA Algorithm

#### 3.3.1. Standard WOA Algorithm

The Whale Optimization Algorithm [17] is a population intelligence optimization algorithm proposed by Mirjalili et al. in 2016. The algorithm has the advantages of simplicity of operation, few parameters to adjust, and the ability to jump out of the local optimum. In the WOA algorithm, the position of each humpback whale represents a feasible solution.

1. Encircling prey:
  The current best candidate solution is assumed to be the target prey or close to the optimal solution. After defining the best candidate solution, other search agents will try to move towards the optimal location and update their positions. The mathematical model is as follows:
  (8) $$D=\left|CX^{*}(t)-X(t)\right|$$
  (9) $$X(t+1)=X^{*}(t)-A\cdot D$$
  where *t* is the current number of iterations; $X^{*}(t)$ denotes the position vector of the optimal solution so far; *X*(*t*) denotes the position vector of the current solution; and *A* and *C* are coefficient variables, calculated by the following equation:
  (10) $$A=2{ar}_{1}-a$$
  (11) $$C=2{ar}_{2}$$
  (12) $$a=2-\frac{2t}{t_{\max}}$$
  where *r* is a random vector in [0, 1]; $t_{\max}$ is the maximum number of iterations.

2. Bubble-net attacking method:
  The whale swims in a spiral motion towards its prey while also shrinking the encirclement. It is assumed that there is a 50% probability of choosing between the shrinking encircling mechanism or the spiral model to update the whale’s position during the optimization process. The mathematical model is as follows:
  (13) $$X(t+1)=\{\begin{array}{c} \begin{array}{cc} X^{*}(t)-A\cdot D & p<0.5 \end{array}\\ \begin{array}{cc} D\prime \cdot e^{bl}\cdot \cos(2\pi l)+X^{*}(t) & p\geq 0.5 \end{array} \end{array}$$
  (14) $$D\prime =\left|X^{*}(t)-X(t)\right|$$
  *D*′ denotes the distance between the current search agent and the current optimal solution; *b* is a constant defining the shape of the logarithmic spiral; *l* is a random number between [−1, 1]; *p* is a random number between [0, 1], i.e., the probability of showing the occurrence of the two behaviors.

3. Search for prey:
  When *|A|* < 1, the whale attacks its prey, demonstrating local search capability. When *|A|* > 1, the WOA algorithm performs a global search for superiority. To ensure that all whales can fully search in the solution space, a search agent is randomly selected, and the positions of other whales are updated according to the randomly selected whale positions, thus achieving a random search.
  (15) $$D=\left|C\cdot X_{rand}-X\right|$$
  (16) $$X(t+1)=X_{rand}-A\cdot D$$
  where $X_{rand}$ denotes the randomly selected whale position vector.

#### 3.3.2. The Binary WOA Algorithm

The standard whale optimization algorithm is proposed for a continuous search space. At the same time, cluster head selection for the deployed UAV nodes is a discrete problem, so a discrete binary optimization algorithm is required to solve it. Currently, many binary swarm heuristics algorithms have been proposed by researchers. Binary algorithms based on swarm-inspired algorithms have been classified, analyzed, and summarized in the literature [18]. In this paper, the discrete binary whale optimization algorithm (BWOA) is considered for CH selection.

According to [19], the main difference between WOA and BWOA is the position update mechanism. In BWOA, the position update is based on a switch between values 1 and 0. The change of the current bit is determined by a probability calculated based on the spiral motion of the humpback whale. The discretization is achieved by mapping the distance between 0 and 1 using a sigmoid function. Where in the probability mapping function [20,21], it is considered that (1) the value should lie in [0, 1] since the transfer function represents the probability of changing position from 0 to 1 and vice versa, and (2) the transfer function should be proportional to the distance between the humpback whale and the prey, i.e., the search agent far from the best search agent should have a higher probability.

1. Shrinking encircling mechanism
  The probability function is calculated as follows:
  (17) $$\gamma_{1}=\frac{1}{1+e^{-10(A\cdot D_{1}-0.5)}}$$
  *A* and $D_{1}$ are calculated using Equations (8) and (10) in the standard algorithm, and γ is theprobability to determine if the bit value should be switched. The position of the search agent is modified as follows:
  (18) $$X(t+1)=\{\begin{array}{c} \begin{array}{cc} \mathbb{C}\left(X(t)\right) & \delta <\gamma_{1} \end{array}\\ \begin{array}{cc} X(t) & \delta \geq \gamma_{1} \end{array} \end{array}$$
  δ is a uniform random number in [0, 1], and ℂ denotes the complementary operation.

2. Spiral update position
  $\gamma_{2}$ can be calculated using the following probability mapping function.
  (19) $$\gamma_{2}=\frac{1}{1+e^{-10(A\cdot D_{2}-0.5)}}$$
  *A* are $D_{2}$ computed by the standard Algorithms (8) and (14). The position in the spiral update position mechanism is calculated as follows:
  (20) $$X(t+1)=\{\begin{array}{c} \begin{array}{cc} \mathbb{C}\left(X(t)\right) & \delta <\gamma_{2} \end{array}\\ \begin{array}{cc} X(t) & \delta \geq \gamma_{2} \end{array} \end{array}$$

3. Search for prey
  The probability is calculated as follows:
  (21) $$\gamma_{3}=\frac{1}{1+e^{-10(A\cdot D_{3}-0.5)}}$$
  *A* are $D_{3}$ computed by the standard Algorithms (8) and (15). As a result, the location of the search agent is updated as follows:
  (22) $$X(t+1)=\{\begin{array}{c} \begin{array}{cc} \mathbb{C}\left(X(t)\right) & \delta <\gamma_{3} \end{array}\\ \begin{array}{cc} X(t) & \delta \geq \gamma_{3} \end{array} \end{array}$$

## 4. Scheme Description

### 4.1. Optimal Number of Cluster Heads

The total network energy consumption is related to the number of clusters. Too few clusters result in higher CH node load, premature energy depletion, and increased cluster reconfiguration. However, dividing too many clusters increases the number of routing hops. It leads to higher transmission delays, so determining the optimal number of clusters can reduce unnecessary network overhead and lower energy consumption. In this paper, the optimal number of clusters is determined by a mathematical model based on coverage and bandwidth balancing.

#### 4.1.1. Coverage Analysis

In this paper, we extend the coverage analysis method in [7] to determine the optimal number of CHs. For any ${UAV}_{i}\in U$ and ${CH}_{j}\in C$, a UAV is covered by a CH if the Euclidean distance Dis(i,j) from UAV node *i* to CH UAV node *j* is less than or equal to the CH perception radius. Usually, UAV nodes choose the nearest CH UAV and join its cluster. Using the binary perception model, $L_{ij}$ is used to represent the connection status of nodes ${UAV}_{i}$ and ${UAV}_{j}$. By calculating the nearest distance $D_{ij}$ from UAV node *i* to CH, if $D_{ij}$ is less than or equal to the CH sensing radius, set $L_{ij}$ = 1; otherwise, $L_{ij}$ = 0.

(23) $$D_{ij}=\min\left\{Dis(i,1),Dis(i,2),\ldots ,Dis(i,K)\right\}$$

(24) $$Dis(i,j)=\sqrt{{\left(x_{i}-x_{j}\right)}^{2}+{\left(y_{i}-y_{j}\right)}^{2}+{\left(z_{i}-z_{j}\right)}^{2}}$$

(25) $$L_{ij}=\{\begin{array}{c} Dis(i,j)\leq R_{j}\\ Dis(i,j)>R_{j} \end{array}$$

It is guaranteed that each node is covered by at least one CH to satisfy the goal that all nodes join the cluster division. In contrast, each UAV node can only belong to one cluster and connect with one CH, so the number of CHs needs to satisfy the following constraints:

(26) $$\sum_{\forall j\in C}L_{ij}=1,\forall i\in U$$

(27) $$\sum_{\forall i\in U}L_{ij}=N-K$$

#### 4.1.2. Bandwidth Analysis

Due to the limited bandwidth of the UAV network communication module, when the number of clusters is too small, the number of nodes in the cluster becomes more, and the data transmission is prone to congestion, and the intra-cluster throughput will be reduced; if the number of clusters is too large, it will lead to higher communication and transmission delay, and the bandwidth is not fully used. Therefore, in order to make full use of the network bandwidth, the intra-cluster and inter-cluster network throughput should be balanced. $B_{1}$ and $B_{2}$ are the intra-cluster and inter-cluster bandwidths, where the number of CMs in the *j*th cluster is $M_{j}$. The optimal number of CHs should satisfy the following bandwidth constraints:

(28) $$\frac{1}{K}\sum_{j=1}^{K}\frac{B_{1}}{\sqrt{M_{j}}}\leq \frac{B_{2}}{\sqrt{K}}$$

(29) $$\sum_{\forall j\in C}M_{j}=N-K$$

Thus the problem of the optimal number of CHs to be solved can be formulated as solving the objective function:

(30) $$\begin{array}{c} \min\sum_{j=1}^{K}j\\ \begin{array}{cc} s.t. & \sum_{\forall j\in C}L_{ij}=1,\forall i\in U \end{array}\\ \begin{array}{cc} & \sum_{\forall i\in U}L_{ij}=N-K \end{array}\\ \begin{array}{cc} & \frac{1}{K}\sum_{j=1}^{K}\frac{B_{1}}{\sqrt{M_{j}}}\leq \frac{B_{2}}{\sqrt{K}} \end{array} \end{array}$$

Constraints: 1. Each cluster member UAV is connected to only one CH, 2. All cluster member UAVs are connected to CH UAVs. 3. Bandwidth balancing is achieved within and between clusters.

### 4.2. Cluster Formation

After determining the optimal number of clusters, the Binary Whale Optimization (BWOA) algorithm is used to dynamically select the optimal cluster head based on the residual energy, distance, and load balancing of the clusters. Then the UAVs are divided into the nearest clusters based on the distance between nodes.

#### 4.2.1. CH Selection Based on BWOA

After cluster formation, the discrete binary whale optimization (BWOA) algorithm is used for cluster head selection with the optimization objectives of reduced energy consumption and load balancing, where the fitness function is defined based on parameters such as residual energy of UAV nodes, intra-cluster distance, inter-cluster distance, and cluster load balancing.

**Figure 2:**
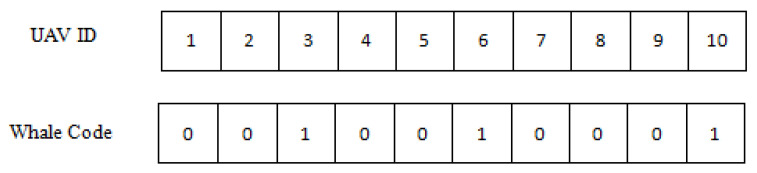
Whale Code.

As shown in Figure 2 below, the whales are binary encoded. Cluster head election is performed for *N* UAVs, each UAV node has a unique ID number, and a whale represents a feasible solution to the cluster head election problem, namely a cluster head election scheme. The position of the whale at a certain moment is an N-dimensional binary vector, and the *i*th bit of the encoded whale is 1, which means that the UAV is elected as a cluster head, and 0 means it is a non-cluster head UAV. The UAVs with IDs 3, 6, and 10 in the figure below are elected as cluster heads.

Fitness Function.

CH nodes are mainly responsible for intra-cluster and inter-cluster communication and data fusion, which will consume more energy than ordinary CM nodes. Higher energy consumption may cause premature failure of nodes and thus lead to degradation of network performance, so the primary factor to consider when selecting a cluster head is to choose a UAV node with high residual energy as CH, and a CH with high residual energy can better maintain the connectivity of the network. The objective function $f_{1}$ of the residual energy is the ratio of the sum of the residual energy of all nodes to the sum of the residual energy of all CHs, which is defined as follows:

(31) $$f_{1}=\sum_{i=1}^{N}E({UAV}_{i})/\sum_{j=1}^{K}E({CH}_{j})$$

where $E({UAV}_{i})$ denotes the residual energy of the *i*th UAV node in the network (*i* = 1, 2, 3, …, *N*) and $E({CH}_{j})$ is the residual energy of the *j*th CH node (*j* = 1, 2, 3, …, *K*). The smaller the value of *f*_1_, the higher the remaining energy of that group of CH nodes.

CM UAV nodes closer to CH nodes can reduce intra-cluster communication energy consumption. $f_{2}$ is the sum of the average intra-cluster CH node distance from all CM nodes for *K* clusters in the network. A smaller value of $f_{2}$ means that the communication energy consumption of that group of nodes as CH is low.

(32) $$f_{2}=\sum_{j=1}^{K}\left(\frac{1}{m}\sum_{i=1}^{m}Dis({UAV}_{i},{CH}_{j})\right)$$

$Dis({UAV}_{i},{CH}_{j})$ is the distance in the cluster and between cluster members.

When nodes send data to the base station, the longer distance may lead to packet loss or delay problems and reduce the reliability of data transmission. $f_{3}$ is the sum of the distances between each CH and the base station, so the smaller the $f_{3}$ value, the more likely the group of nodes will be selected as CH UAV. The function is defined as follows:

(33) $$f_{3}=\sum_{j=1}^{K}Dis({CH}_{j},BS)$$

An unbalanced load also degrades the communication quality of the network. CHs with high load will consume energy faster and reduce the overall performance of the network, so the closer the number of cluster members, the more balanced the energy consumption among CHs and the longer the network lifetime. $f_{4}$ is the sum of the degree of difference between the individual cluster sizes and the average cluster size, and the function is defined as follows:

(34) $$f_{4}=\sum_{j=1}^{K}\left|\frac{N}{K}-{Cnum}_{j}\right|$$

where ${Cnum}_{j}$ is the size of cluster *j*. The smaller the $f_{4}$ value, the more balanced the network load.

Therefore, the CH selection takes into account the parameters of residual energy, intra-cluster distance, inter-cluster distance, and cluster load balancing and defines the CH selection adaptation function as follows:

(35) $$\mathrm{Fitness}=w_{1}\times f_{1}+w_{2}\times f_{2}+w_{3}\times f_{3}+w_{4}\times f_{4}$$

where $w_{1}+w_{2}+w_{3}+w_{4}=1$, $w_{1},w_{2},w_{3},w_{4},$ are the weight parameters between (0, 1).

The pseudo-code for CH selection based on the BWOA algorithm is given in Algorithm 1. After completing CH selection, for all non-CH UAVs, the distance between the UAV and all CH nodes within its communication range is calculated. Then the nearest cluster is selected to join. Algorithm 2 gives the cluster formation pseudo-code.
**Algorithm 1: CH Selection Based on BWOA****Input:**${UAV}_{i}$, *i* = 1, 2, …, *N*.**Output:** Cluster head ${CH}_{j}$, *j* = 1, 2, …, *K*./* Initialization phase*/1: Initialize the whale population X=[X1,X2,…,XN].2: Initialize iteration t=1, maximum number of iterations tmax.3: Calculate the fitness of each whale.$4:\;\mathrm{Choose}\;\mathrm{the}\;\mathrm{best}\;\mathrm{solutions}\;X^{*}(t)$/* Computation*/5: **while**
$(t<t_{\max}$), **do**6:     **for** each search agent, do7:         Update *A, C* and *a* by (10)–(12).8:         **if**
*p* < 0.5 then9:            **if**
|A|<1
**then**$10:\;\;\;\;\;\;\;\;\;\;\;\;\;\;\;\mathrm{Update}\;D_{1}$$\;\mathrm{by}\;(8)\;\mathrm{and}\;\gamma_{1}$ by (17).11:               Update the position *X* based on (18).12:            **else**$13:\;\;\;\;\;\;\;\;\;\mathrm{Select}\;a\;\mathrm{random}\;X_{rand}$$\;\mathrm{and}\;\mathrm{update}\;D_{3}$ by (15) and (16).$14:\;\;\;\;\;\;\;\;\;\mathrm{Update}\;\gamma_{3}$ by (21) and the position *X* by (22).15:           **end if**16:        **else**17:            Update D2 by (14) and γ2 by (19).18:            Update the position *X* by (20).19:        **end if**20:     **end for**21:     Calculate the fitness of each search agent by (39).$22:\;\;\;\;\;\mathrm{Update}\;X^{*}(t)$ of the best search agent.23:     *t* = *t* + 124: **end while**25: return CHs


**Algorithm 2: Cluster Formation**
**Input:**${UAV}_{i}$, *i* = 1, 2, …$,\;N,\;{CH}_{j}$, *j* = 1, 2, …, *K*.**Output:** Cluster ${CL}_{j}$, *j* = 1, 2, …, *K*.1: **for** each ${UAV}_{i}$
**do**2:   **Compute** (the distance between the UAV and all CH within its communication range)3: **if** the UAV has a minimum distance from ${CH}_{j}$
**then**

$4:\;\;\;\;\;\;\;\mathrm{UAV}\;\mathrm{joins}\;\mathrm{the}\;{CL}_{j}$

5:   **end if**6: **end for**$7:\;\mathrm{Return}\;{CL}_{j}$, *j* = 1, 2, …, *K*.

#### 4.2.2. Cluster Management Phase

After the cluster division is completed, as the UAV nodes consume energy and move, the nodes within the cluster may not be sufficient to continue to act as cluster heads, and the cluster needs to be re-divided. The following cases require cluster maintenance:

1. Set the energy threshold for CH, and periodically check the node energy. If it is lower than this value or CH leaves the cluster, then perform cluster maintenance and re-execute Algorithms 1 and 2.
2. If the CM leaves the cluster, the node is removed from the list of members of that cluster.

### 4.3. Routing Mechanism

For the UAV cluster communication relay routing problem, intra-cluster and inter-cluster communication are considered, respectively. For intra-cluster communication: first, look up the neighbor table. If the destination node is in its neighbor table, then communicate directly, otherwise forward to the cluster head to communicate with the destination node; for inter-cluster communication: select the next hop node based on the weighting function of the remaining energy and distance of the UAV node, so that the relay node load is balanced, thus improving communication efficiency and reducing communication cost. Inter-cluster communication path selection function:

(36) $${path}_{ij}=\frac{E_{j}}{Dis(i,j)}$$

Ej denotes the residual energy of node *j*; Dis(i,j) denotes the distance between nodes *i* and *j*. The node with the highest residual energy and the shortest distance is preferably considered for next-hop routing.

## 5. Simulation Results and Analysis

In this section, we perform experiments using MATLAB to evaluate the performance of the BWOA algorithm-based clustering scheme. Firstly, we introduce the settings of simulation parameters and visually analyze and compare the benefits of cluster clustering and compare the analysis with BPSO [22], GWO [23], and K-means [24] based schemes in terms of cluster building time and average cluster lifetime, energy consumption, and UAV node survival rate.

The simulation parameters are set as shown in Table 2. The simulation scene is a three-dimensional space of 2000 m × 2000 m × 500 m, and the parameters in the energy consumption model are set as follows: $E_{elec}=50\;{\mathrm{nJ}/\mathrm{bit}/m}^{2}$; $\varepsilon_{fs}=100\;\mathrm{pJ}/\mathrm{bit}$; $\varepsilon_{amp}=0.01\;{\mathrm{pJ}/\mathrm{bit}/m}^{4}$; UAV mass $m_{u}=0.7\;\mathrm{kg}$, gravitational acceleration $g=9.8\;{m/s}^{2}$, radius and number of wings $r_{w}=0.2\;m$ and $n_{w}=4$, respectively, and air density $\rho_{a}=1.29\;{\mathrm{kg}/m}^{3}$. The flight speed is 10 m/s–30 m/s. The data link layer model of FANET uses IEEE 802.11 protocol [25] with a link bandwidth of 2 Mbps.

*A.*  *Benefits of Clustering*

**Figure 3:**
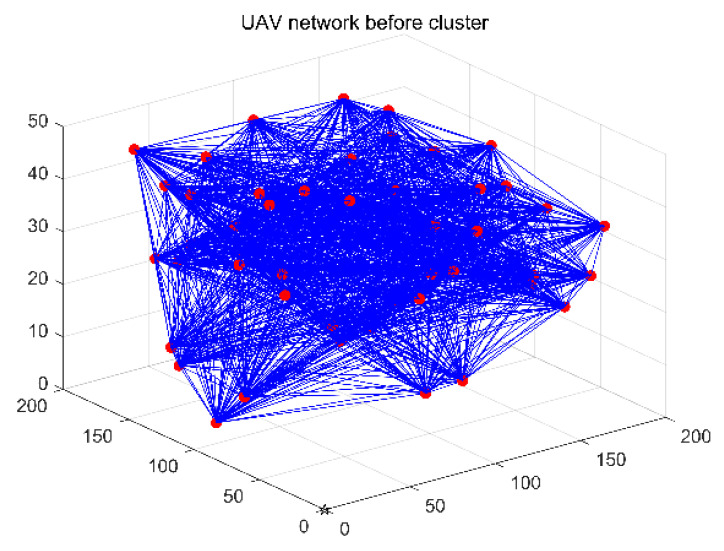
UAV network before cluster.

**Figure 4:**
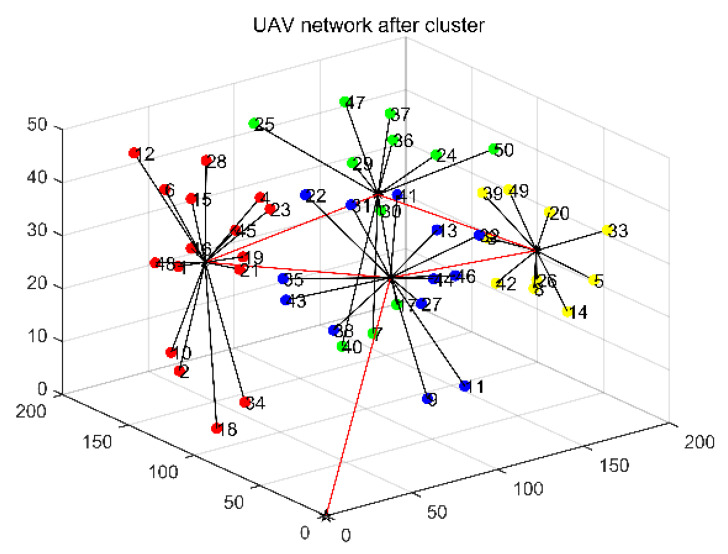
UAV network after cluster.

Cluster management can reduce the amount of network communication, reduce overhead, and extend the network’s lifetime. The visual comparison analysis from the above figure shows that: as shown in Figure 3, before dividing the clusters, all nodes try to communicate with each other, and the network overhead increases exponentially. In Figure 4, after dividing the clusters through cluster header data fusion, nodes transmit packets to CH, which are forwarded by CH to the destination node or base station, which reduces the overhead, reduces the communication of nodes within the network, and thus improves the network lifetime.

*B.*  *Cluster Building Time*

**Figure 5:**
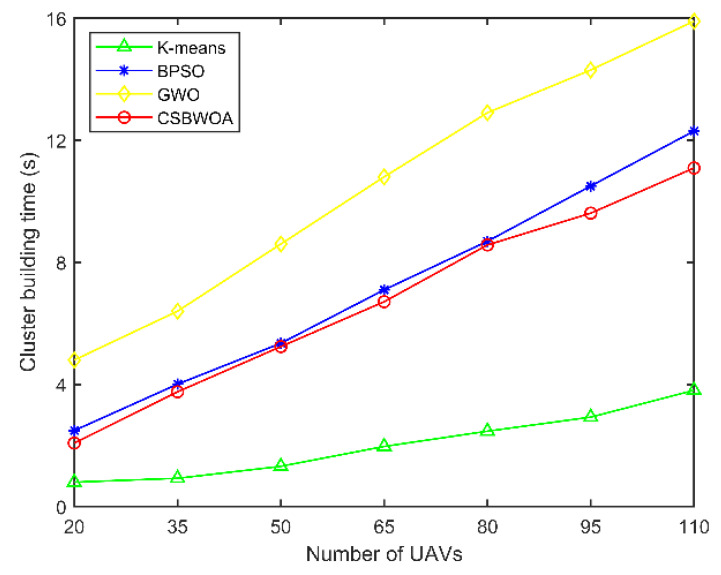
Cluster building time vs. Number of UAVs.

Cluster building time is the time spent to execute the clustering algorithm to select CH and form clusters and represents the computational complexity of the algorithm. The shorter the build time, the lower the energy consumption. Figure 5 depicts the relationship between cluster building time and the number of UAVs, from which it can be seen that the cluster building time increases with the number of UAVs in the network. The CSBWOA is slightly better than BPSO. The K-means-based clustering scheme produces only one solution and updates to the optimal global solution sequentially, so its cluster building time is shorter. Although CSBWOA takes slightly longer to build, its optimal CH selection has good performance in improving the network performance.

*C.*  *Cluster Lifetime*

**Figure 6:**
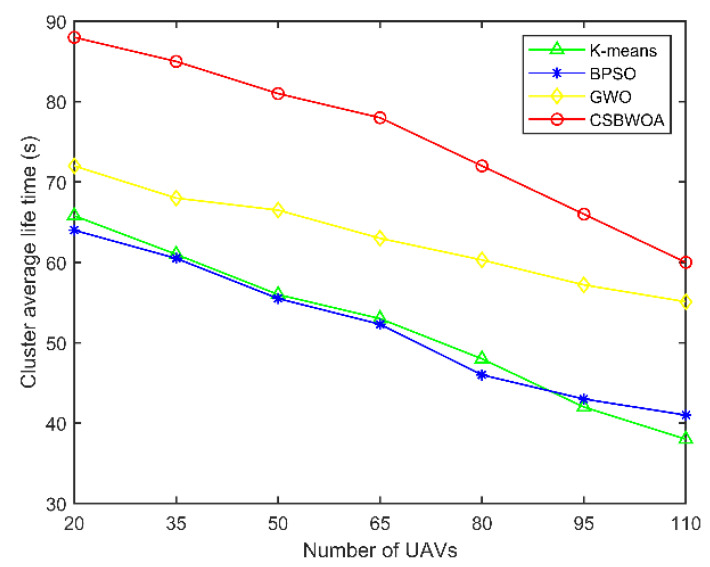
Cluster average lifetime vs. Number of UAVs.

The cluster lifetime is the time interval between the election of CHs and cluster formation and the next re-election of CHs to divide the cluster. The longer the cluster lifetime, the fewer the number of cluster reconfigurations. Figure 6 depicts the effect of the number of UAVs on the average cluster lifetime, which gradually decreases as the number of UAVs increases. However, it can be seen from the figure that the average cluster lifetime of the proposed CSBWOA is longer because the proposed scheme cluster division is more reasonable. The optimal CH is selected by considering energy consumption and distance and cluster load balancing, which reduces the number of cluster reconfigurations and maintains a stable cluster structure, resulting in a longer cluster lifetime.

*D.*  *Energy Consumption and Number of Rounds*

**Figure 7:**
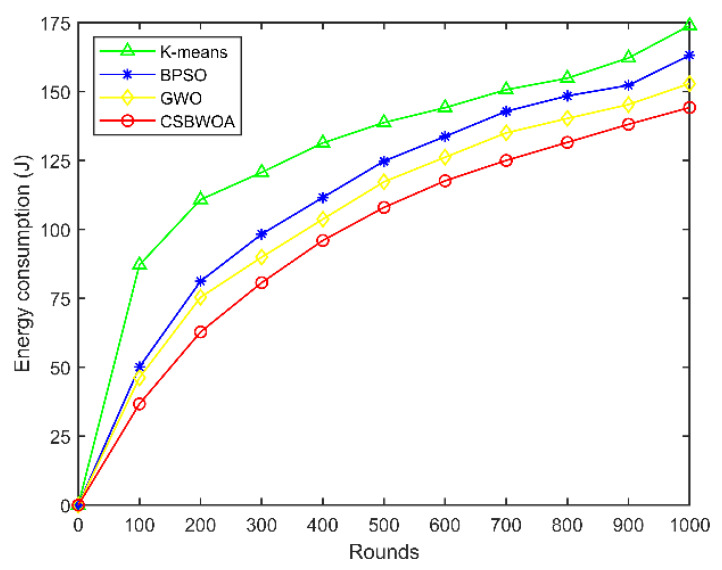
Energy consumption vs. Rounds (Number of UAVs is 35).

Figure 7 depicts the variation curves of the energy consumption of the three algorithms with the number of rounds when the number of UAVs is 35. It can be seen from the figure that as the number of rounds increases, the energy consumption of CSBWOA is lower compared with the other algorithms. This is because the residual energy of UAVs and the overall cluster load balancing are considered in the optimal CH selection; the cluster maintenance policy is also set to maintain the cluster stability, which avoids frequent cluster reconfiguration to a certain extent and thus consumes less energy.

**Figure 8:**
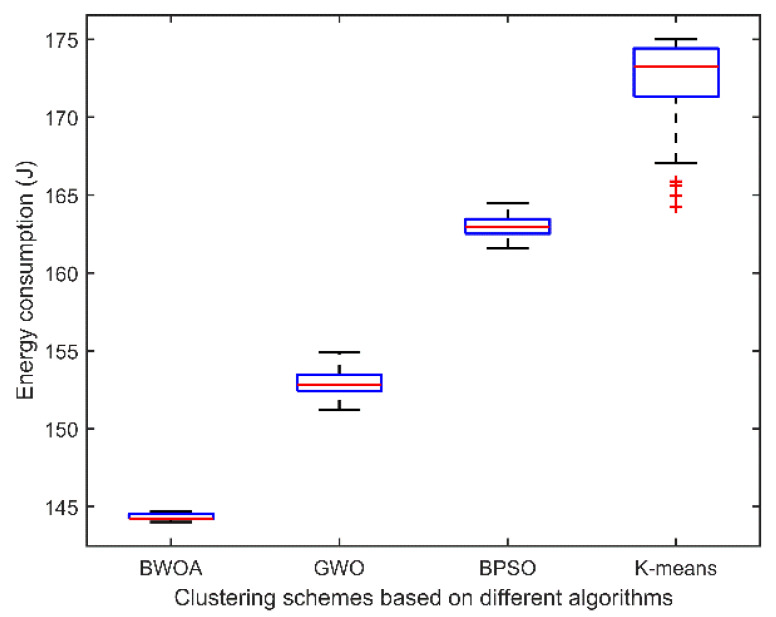
Boxplot of energy consumption for clustering schemes based on different algorithms (Number of UAVs is 35 and number of rounds is 1000).

The dispersion of the energy consumption values for all clustering schemes independently simulated 30 times for the scenario with the number of UAVs of 35 is shown in Figure 8. The energy consumption of the BWOA-based clustering scheme is lower among all schemes. And it can be seen from the boxplot that the proposed scheme in this paper has the smallest standard deviation compared with the clustering schemes based on other algorithms.

*E.*  *Node Survival Rate and Number of Rounds*

**Figure 9:**
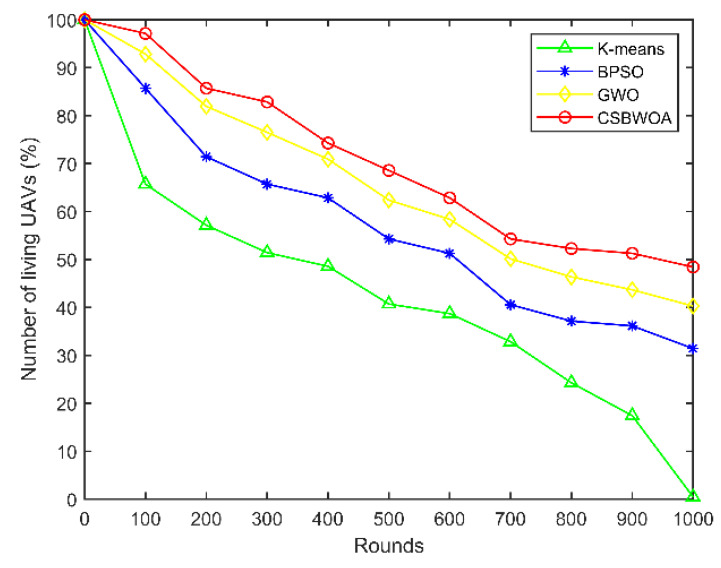
Number of living UAVs vs. Rounds (Number of UAVs is 35).

The UAV survival rate is the ratio of the number of surviving UAVs to the total number of UAVs. Figure 9 shows the results of node survival rate comparison of different algorithms when the number of UAV nodes is 35. It can be seen from the figure that the survival rate of UAVs in the CSBWOA is higher than in other algorithms as the number of rounds increases. This is because, due to the optimal CH selection, the algorithm proposed in this paper can better balance the energy consumption of UAVs, which prolongs the lifetime of UAVs and thus has a higher survival rate.

## 6. Conclusions

In this paper, we propose a clustering scheme based on the binary whale optimization algorithm for the energy consumption problem in FANET. Firstly, the optimal number of CHs is calculated under the conditions of coverage demand and bandwidth balancing. Then the fitness function for CH selection is designed by considering the energy of UAVs, inter-cluster and intra-cluster distances, and the load balancing parameter indexes of clusters, and the optimal CHs are selected based on the BWOA algorithm. Then, UAVs are divided into the nearest clusters according to the distance. The clusters are maintained efficiently according to the set maintenance strategy. The node with the highest energy at the closest distance is preferred as the next hop when UAVs communicate. The MATLAB simulation experiments prove that the proposed scheme in this paper outperforms the BPSO-based and K-means-based clustering schemes in terms of network energy consumption and cluster survival. The clusters are efficiently maintained according to the set maintenance policy. The node with the highest energy at the closest distance is preferred as the next hop when the UAV communicates. It is proved by MATLAB simulation experiments that the proposed scheme in this paper outperforms the BPSO-based and K-means-based clustering schemes in terms of network energy consumption and cluster lifetime.

In the future, hybrid bionic optimization algorithms can be considered for CH selection to solve some existing problems in bionic optimization algorithms that are easy to fall into local optimality, to select the CH with optimal performance so that the network energy consumption is lower and the network survival period is longer.

## Figures and Tables

**Table 1:** Summary and comparison of CSBWOA with other clustering schemes.

Proposals	Selection of Cluster Head			Cluster Building Time	Cluster Lifetime	Energy Consumption	Survival Rate
	Distance	Energy	Load Balancing				
EALC [6]	Y	Y	N	Y	Y	Y	N
MLSC [7]	Y	N	N	N	N	N	N
SAD-CA [8]	N	N	N	N	Y	Y	N
IWLC [9]	Y	Y	Y	N	Y	Y	N
BICSF [10]	N	Y	N	Y	Y	Y	N
CRSF [11]	Y	Y	N	N	Y	Y	N
SIC [12]	Y	Y	N	Y	Y	Y	Y
BIC [13]	Y	Y	Y	Y	Y	Y	Y
DCM [14]	Y	Y	N	N	N	Y	N
CSBWOA	Y	Y	Y	Y	Y	Y	Y

**Table 2:** Simulation parameters.

Parameters	Value
Network simulation	MATLAB
Simulation area	2000 m × 2000 m × 500 m
Number of UAVs	20~110
Number of ground stations	1
Transmission frequency	2.4 GHz
Communication standard	IEEE 802.11n
UAV transmission range	300 m
Minimum distance between UAVs	10 m
Initial energy	5 J
Rounds	1000
Data packet	50 KB
Number of iterations	Variable
Number of runs	30

## Data Availability

Not applicable.
